# Linkage disequilibrium of evolutionarily conserved regions in the human genome

**DOI:** 10.1186/1471-2164-7-326

**Published:** 2006-12-28

**Authors:** Mamoru Kato, Akihiro Sekine, Yozo Ohnishi, Todd A Johnson, Toshihiro Tanaka, Yusuke Nakamura, Tatsuhiko Tsunoda

**Affiliations:** 1 SNP Research Center, RIKEN, Yokohama, Japan; 2 Human Genome Center, Institute of Medical Science, University of Tokyo, Tokyo, Japan

## Abstract

**Background:**

The strong linkage disequilibrium (LD) recently found in genic or exonic regions of the human genome demonstrated that LD can be increased by evolutionary mechanisms that select for functionally important loci. This suggests that LD might be stronger in regions conserved among species than in non-conserved regions, since regions exposed to natural selection tend to be conserved. To assess this hypothesis, we used genome-wide polymorphism data from the HapMap project and investigated LD within DNA sequences conserved between the human and mouse genomes.

**Results:**

Unexpectedly, we observed that LD was significantly weaker in conserved regions than in non-conserved regions. To investigate why, we examined sequence features that may distort the relationship between LD and conserved regions. We found that interspersed repeats, and not other sequence features, were associated with the weak LD tendency in conserved regions. To appropriately understand the relationship between LD and conserved regions, we removed the effect of repetitive elements and found that the high degree of sequence conservation was strongly associated with strong LD in coding regions but not with that in non-coding regions.

**Conclusion:**

Our work demonstrates that the degree of sequence conservation does not simply increase LD as predicted by the hypothesis. Rather, it implies that purifying selection changes the polymorphic patterns of coding sequences but has little influence on the patterns of functional units such as regulatory elements present in non-coding regions, since the former are generally restricted by the constraint of maintaining a functional protein product across multiple exons while the latter may exist more as individually isolated units.

## Background

Linkage disequilibrium (LD) is non-random association between alleles at different loci and helps us to reconstruct the genetic history of human populations and to improve our understanding of the biological processes of recombination and natural selection [1]. LD also helps association studies to identify haplotypes that are linked to disease-causing variations. Early studies of LD focused on small sets of genes, such as the HLA genes [2] or the growth hormone gene cluster [3]. Recently, large-scale genotyping studies [1,4-6] have investigated the genomic patterns of LD in the human genome and have found considerable variation in its values, even for SNP pairs that are separated by identical physical distances. Some studies have tried to associate this variation with sequence features existing in the genome and found that genic or exonic regions are associated with strong LD in human populations. For example, extended LD regions are significantly overpopulated with SNPs located in genic or coding regions [5], and LD is stronger between exonic variants within a gene compared with intronic or intergenic SNPs [6]. The recent International HapMap Project also revealed an excess number of genes with strong LD on a genomic scale [7]. These findings can be explained by the previous hypothesis that purifying selection leads to an increase of LD [8]. This basic hypothesis in turn suggests that LD might be stronger in regions conserved among species than in non-conserved regions, since regions exposed to purifying selection tend to be conserved over evolutionary time.

In the present study, using the complete human/mouse sequences and polymorphic data from the HapMap Project, we unexpectedly observed that LD was significantly weaker in conserved regions than in non-conserved regions. A similar tendency was recently reported in a companion paper of the HapMap project [9]. To investigate this inconsistency between the predicted and observed tendencies, we examined the possibility that the relationship between LD and conserved regions is distorted by other sequence features, such as physical distance, genic content, GC/CpG frequency, or chromosomal location. However, these features were independent of the weaker LD tendency in conserved regions. Finally, we found that LD patterns carried by interspersed repeats were associated with this discrepancy. To precisely understand the relationship between LD and sequence conservation, we removed the effect of repetitive elements from the LD patterns, and found that although the previous hypothesis is partly correct, the reality is more complex than expected. That is, sequence conservation itself is not well associated with the degree of LD; however, on conserved coding regions only, it is related to a strong degree of LD. The results of our detailed analysis of the LD tendency in conserved regions imply that selective force produces a more complicated tendency in polymorphic patterns that varies according to the long-range or short-range functionality of DNA sequences.

## Results

### LD within conserved regions

**Figure 1:**
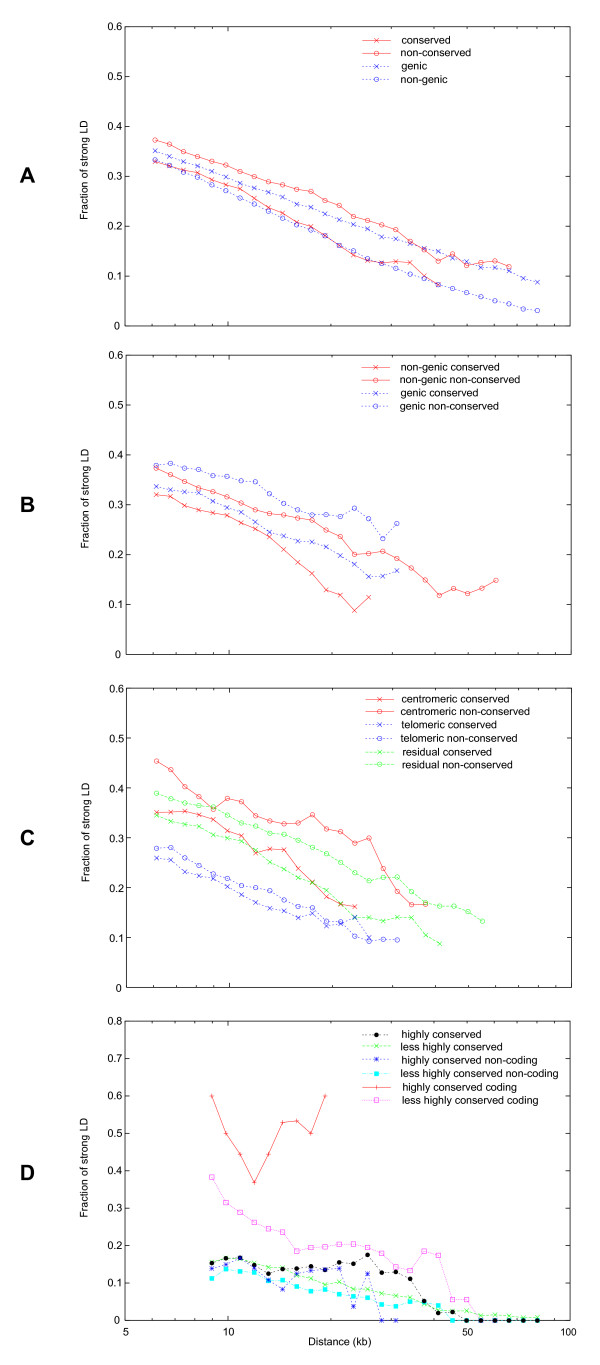
**A moving average of the fraction of complete or nearly complete LD (*r*^2 ^> 0.8) versus distance between SNPs**. All panels are those for CEU. See Additional file 1 for CHB, JPT, and YRI, which show the same tendency. (A) Plots of LD within DNA sequences conserved between the human and mouse genomes (in red with Xs), non-conserved regions (regions other than conserved ones; shown in red with circles), genic regions (in blue with Xs), and non-genic regions (in blue with circles). (B) Plots of LD within intersections of non-genic regions with conserved (in red with Xs) and non-conserved (in red with circles) regions, and of genic regions with conserved (in blue with Xs) and non-conserved (in blue with circles) regions. (C) Plots of LD within intersected regions of centromeric regions (the 10% definition, we only show plots in the 10% definition because of the same tendency in the 5% definition) with conserved (in red with Xs) and non-conserved (in red with circles) regions, of telomeric regions with conserved (in blue with Xs) and non-conserved (in blue with circles) regions, and of the residual regions (neither centromeric nor telomeric) with conserved (in green with Xs) and non-conserved (in green with circles) regions. (D) LD fractions for SNP pairs within highly conserved and less highly conserved regions (black and green), highly and less highly conserved non-coding regions (blue and light blue), and regions enriched (>20% in the bases) with highly and less highly conserved coding regions (red and pink). We selected only regions where the proportion of repeats was <20%, and since after this adjustment we found outliers of LD related to extreme GC-content, we further selected regions where the GC-content was 45–65%.

We calculated pairwise *r*^2 ^and |*D*'| values within conserved and non-conserved regions across the human genome and found that conserved regions contained lower proportions of SNP pairs that were in complete or nearly complete LD (*r*^2 ^> 0.8, |*D*'| > 0.9) when calculated as a function of physical distance (Fig. 1A for CEU and Additional file 1 for CHB, JPT, and YRI). A permutation test confirmed the significance of this observation (*p *< 10^-4 ^for all 10 kb bins of distance up to 40 kb; see Methods). We confirmed that allele frequencies had no effect on this result (data not shown). Since all results described here and below had the same tendencies for both *r*^2 ^and |*D*'|, we show only the *r*^2 ^results. We further checked the result by fine-scale recombination rates from the HapMap data [7] and found a higher recombination rate (1.41 cM/Mb on average) in conserved regions than that (1.26 cM/Mb) in non-conserved regions. This result is consistent with the LD results, since, in general, lower LD values are widely known to be related to higher recombination rates [8,10,11].

The finding of lower LD in conserved regions is inconsistent with the hypothesis that purifying selection increases the extent of LD [8]. Therefore, we first considered the possibility that the unexpected decrease of LD in conserved regions (i.e., the increase of LD in non-conserved regions) was distorted by the presence of genes, since genic regions had previously been shown to exhibit increased LD [5]. For that purpose, we took intersected regions of conserved/non-conserved regions with genic/non-genic regions, and generated datasets for four classes of regions: conserved genic, conserved non-genic, non-conserved genic, and non-conserved non-genic. Figure 1B (and Additional file 1) shows that conserved regions still possessed lower proportions of SNP pairs in strong LD compared to non-conserved regions for both genic and non-genic classes. Thus, gene content does not account for this effect. Next, since centromeric regions show stronger LD than telomeric regions [6,12], we also checked the possible involvement of chromosomal location by intersecting conserved and non-conserved regions with telomeric, centromeric, and other residual regions (see Methods). Figure 1C (and Additional file 1) shows the same tendency for all centromeric, telomeric, and residual regions. This result suggested that the weak LD in conserved regions was independent of chromosomal location.

In view of these results, we considered whether other factors, such as GC-content or CpG dinucleotides, may have been involved in the weak LD in conserved regions, because it was recently found that GC-content is associated with weak LD on a genomic scale [7,9]. However, GC-content and CpG dinucleotides are unlikely to account for the observed LD differences, since the proportions of their bases in conserved regions were almost equal to those in non-conserved ones (Additional file 2). To verify this, we executed permutation tests (see Methods), and found that even when the effect of these sequence features was subtracted from LD, the adjusted LD in conserved regions was still significantly weaker than that in non-conserved ones (*p *< 10^-4^).

### The influence of repetitive elements

Next, we considered whether the weak LD tendency in conserved regions might be related to a lack of interspersed repeats in these regions, since interspersed repeats were recently reported to be related to strong LD on a genomic scale [7,9]. We found that the proportion of the total number of bases in repeats within conserved regions was half of the proportion found within non-conserved ones (Additional file 2), as previously observed [13]; this was probably because local rates of neutral variation may be low in conserved regions [13] or because selective pressure working around conserved regions may have excluded repetitive elements that would cause deleterious changes in the genome, such as changes in a gene's structure [14]. Indeed, our permutation tests showed that, after subtracting the effect of repeats from LD by regression, we no longer observed any significant difference in LD between conserved and non-conserved regions (*p *= 0.522). We confirmed these results by partial correlation analysis (Additional file 3). These findings suggest that the lack of repetitive elements accounts for weak LD in conserved regions. Among the several types of repeats, LINE/L1s had the largest regression coefficient in the regression analysis between LD and the proportion of bases contained in repeats (Additional file 2), the smallest proportion of bases in conserved regions compared to non-conserved ones (Additional file 2), and the largest total number of bases in the human genome (Additional file 2). Therefore, L1s appeared to mostly account for the weak LD in conserved regions.

Since we found that repetitive elements are strongly associated with weaker LD in conserved regions, we adjusted for the base-pair proportion of repeats as well as GC-content; the latter due to outliers of LD related to extreme GC-content after the repeat adjustment. We then compared the LD levels in highly conserved regions with those in less highly conserved regions. We expected that highly conserved regions would have stronger LD because the selective pressure on these regions was considered to be stronger. However, unexpectedly, we found no enhancement of the strong LD fraction within highly conserved regions compared to less highly conserved regions (Fig. 1D and Additional file 1). We then classified these regions into two groups, those enriched with coding sequences and those enriched with non-coding sequences. As a result, we found that regions enriched with highly conserved coding sequences had stronger LD than regions enriched with less highly conserved coding sequences. Meanwhile, no difference in LD was found between highly conserved non-coding regions and less highly conserved non-coding regions. To further confirm these results, we used fine-scale recombination rates from the HapMap data [7] and calculated average recombination rates for the same regions (see Methods). This method revealed a similar tendency, with a somewhat smaller recombination rate shown in highly conserved non-coding regions than in less highly conserved non-coding regions, while the difference was far greater between highly and less highly conserved coding rich-regions (Table 1). These results suggest that purifying selection that works on evolutionarily conserved regions surely increases the LD level in a series of coding sequences; however, it does not do so in non-coding sequences, as discussed below.

## Discussion

Throughout the evolutionary history of a population, a variety of factors influence the LD level, such as recombination, mutation, genetic drift, natural selection, and demographic events [7,8,11,15]. Among these factors, natural selection is considered to generally increase the degree of LD, though there are stochastic fluctuations in individual cases. There are two primary routes for selection to increase LD [8]. The first is a hitchhiking effect (also known as a selective sweep), in which an entire haplotype with an advantageous variant is rapidly selected to high frequency or even fixation [8,15], leading to a high degree of LD carried by the selected haplotype. This occurs in the process of positive (adaptive) selection. Purifying (negative) selection against deleterious variants can also increase LD, as the deleterious haplotypes are swept from the population [8]. The second route is epistatic selection for combinations of alleles at multiple loci [6,8], in which natural selection may favor or may not favor certain combinations of alleles that work synergistically. Recent studies [1,5,7] on LD patterns by large-scale genotyping datasets have demonstrated that LD in genic regions is strong at sizes roughly up to 100 to 200 kb. Because most genes are exposed to purifying (not positive) selection, these studies illustrate that the overall effect of purifying selection is to increase the degree of LD. This in turn suggests that the degree of LD may be strong in regions that are evolutionarily conserved between distantly related species, e.g., humans and mice, because it is evolutionarily conserved regions that remain unchanged by purifying selection [16].

However, a recent HapMap companion paper [9] reported that, although base-pairs in regions conserved between the human and mouse genomes were associated with low LD when sequence features were analyzed individually, the sequence conservation was not identified as an important predictor of LD in a multiple linear regression analysis. Consistent with these findings, although we initially found that conserved regions showed low LD levels, after consideration of sequence features one-by-one, we eventually determined that this relationship between LD and conserved regions was distorted by the lack of repetitive elements in such regions.

Thus, we excluded the effect of repetitive elements (and GC-content as well) in order to determine the relationship between LD and conserved regions. However, the result was not as simple as expected. We did not see a strong association between the level of LD and the degree of conservation in overall conserved regions but observed that strong LD was related to strong conservation in conserved coding regions. In addition, the LD level was not strongly related to sequence conservation in conserved non-coding regions. Because it has been demonstrated that even in non-coding regions, conserved regions include more functionally important segments, such as regulatory elements, than non-conserved regions [17,18], conservation thus seems to indicate selective constraint even in non-coding regions. Taking this into account, one interpretation of our results is that selective force works differentially between coding and non-coding regions. Purifying selection works on the function of exons' final protein products and may not allow frequent recombination between sequential series of coding sequences, which leads to strong LD in these sequences. Meanwhile, the similar LD levels in highly and less highly conserved non-coding regions may be explained by the independence of functional units, such as regulatory elements, present in those non-coding regions. That is, just individual alleles in conserved non-coding regions may be exposed to selective pressure; therefore, they may more often accept recombination between them. Alternatively, our results may suggest that conservation does not indicate selective constraint only for non-coding regions since non-coding regions might include too much noise unlike coding-regions, which are by definition functional.

## Conclusion

Following the previous hypothesis that purifying selection increases the extent of LD, we examined whether LD was actually lower in evolutionarily conserved regions and attempted, by considering one potential factor at a time, to determine if a third factor may distort LD in conserved regions. We found that this tendency was associated with a lack of repetitive elements in those regions. We then showed that after correcting for the effect of repeat abundance, the degree of conservation itself was not strongly associated with the extent of LD in non-coding regions, but it was associated with LD in coding regions, which suggested that the effect of purifying selection on LD was more complex than expected from the previous hypothesis. This can be explained by the idea that natural selection works on the function of conserved exons' final protein products, while it works independently on the constituent alleles of conserved functional units in non-coding regions. In summary, purifying selection may prominently increase the extent of LD only when regions between alleles contain sequentially meaningful segments, such as segments translated into proteins. As we demonstrated, in-depth analyses are needed to elucidate the relationship between LD and sequence features. By means of such analyses, the LD patterns of the human genome may help to clarify the biological processes of recombination, mutation, and natural selection during the evolutionary history of human populations.

## Methods

### Detection of conserved regions

We downloaded the human (build 34) and mouse (build 32) genomic sequences from NCBI and followed a previously described procedure [19] to identify orthologous regions of the human and mouse genomes. We used BlastZ [19] with parameters C = 2, T = 1, K = 3000 to align human and mouse genomic sequences, and obtained alignments between the human and mouse. For overlapping alignment regions, we formed single contiguous regions. For example, when three regions started from coordinates 1 to 10, 5 to 15, and 20 to 30, respectively, we merged the former two regions into one region that started from 1 to 15. We used these contiguous alignment regions as conserved regions, which occupy 44% (1,255,655,305/2,818,767,476 bases, excluding the Y chromosome and gaps between contigs) of the human genome, which is almost the same percent (roughly 40%) as that detected by the previous study [20] of the mouse genome using BlastZ. For other genomic regions described below, we also formed single contiguous regions from any overlapping regions.

### Plot of LD versus distance

To calculate LD, we downloaded genotype data (Release 16a) from the International HapMap Project [4] website. The samples were derived from 90 individuals in Utah, USA, from the Centre d'Etude du Polymorphisme Humain collection (CEU); from 45 Han Chinese in Beijing, China (CHB); from 44 Japanese in Tokyo, Japan (JPT); and from 90 Yoruba in Ibadan, Nigeria (YRI). The datasets of the four groups were treated independently to calculate linkage disequilibrium (LD). For each population, we considered a bi-allelic SNP to be validated in LD calculation as follows: 1) if the genotype data showed no significant deviation from Hardy-Weinberg equilibrium (Fisher's exact *p *> 0.001); 2) if the minor-allele frequency was greater than 0.2; 3) if the Mendelian inconsistency equaled zero; 4) if the position was not found at multiple chromosomal locations; 5) if the position was not located within a repeat element.

Using Haploview [21], we calculated pairwise *r*^2 ^and |*D*'| for all possible pairs of validated SNPs that were separated by distances of less than 100 kb on the same contigs. We selected *r*^2 ^(and |*D*'|) values of SNP pairs both located within specified regions (e.g., conserved regions), and placed them into window bins according to predetermined ranges of distance between SNPs [6]. For a data point at position *x *in the plot figure (Fig. 1), we set the range of each sliding window from *k*^(-1/2)^*x *to *k*^(1/2)^*x *(corresponding to the range from log_10_*x*-(1/2)log_10_*k *to log_10_*x*+(1/2)log_10_*k *on a log scale), where *k *= 1.5, and we set the data point of the next sliding window at position *lx *(corresponding to log_10_*x*+log_10 _*l *on a log scale), where *l *= 1.1. Within each sliding window, we calculated the frequency of complete or nearly complete LD (*r*^2 ^> 0.8, |*D*'| > 0.9), and plotted the data point only when the sample size of those LD values was 100 or more (Fig. 1A, B and C), or 10 or more (Fig. 1D). In permutation tests, we randomly shuffled LD values of conserved and non-conserved regions (for each of the 0–10, 10–20, 20–30, 30–40 kb windows) 10,000 times, and each time we calculated a ratio of the two strong LD fractions of randomized conserved and non-conserved regions to get a *p*-value.

### Datasets intersected with large-scale genomic features

We extracted gene positions from the NCBI Build 34 seq_gene.md mapview annotation file and created datasets consisting of conserved/non-conserved regions intersected with genic/non-genic regions. We also produced datasets involving conserved/non-conserved regions intersected with telomeric, centromeric, and other residual genomic regions. Because it is difficult to strictly define telomeric or centromeric regions, for this intersection we used two definitions (5% or 10%) of distances from the ends of the chromosomal arms distal and proximal to the centromere.

### Highly conserved regions

We downloaded a table (mouse net table) containing coordinates of conserved regions between the human and mouse genomes from the UCSC genome browser website to define highly and less highly conserved regions. We iteratively adjusted a score parameter that was proportional to the sequence identity to obtain two sets of conserved regions that occupied approximately 5% and 40% of the genome, corresponding respectively to highly conserved regions and less highly conserved regions [20].

### Regression analysis and permutation tests

We undertook a regression analysis to evaluate quantitatively how LD is influenced by a given sequence feature for each population. We regressed *r*^2 ^values adjusted by the physical distances with base-pair proportions of each sequence feature within SNP pairs, irrespective of categories of conserved and non-conserved regions. We first regressed observed *r*^2 ^values with the observed physical distances between SNPs using a model explicitly dependent on the distance as described below and obtained *r*^2 ^values that were expected from the distances:

E(r2)=∑n=03an⋅ln,
 MathType@MTEF@5@5@+=feaafiart1ev1aaatCvAUfKttLearuWrP9MDH5MBPbIqV92AaeXatLxBI9gBaebbnrfifHhDYfgasaacH8akY=wiFfYdH8Gipec8Eeeu0xXdbba9frFj0=OqFfea0dXdd9vqai=hGuQ8kuc9pgc9s8qqaq=dirpe0xb9q8qiLsFr0=vr0=vr0dc8meaabaqaciaacaGaaeqabaqabeGadaaakeaacqWGfbqrcqGGOaakcqWGYbGCdaahaaWcbeqaaiabikdaYaaakiabcMcaPiabg2da9maaqahabaGaemyyae2aaSbaaSqaaiabd6gaUbqabaaabaGaemOBa4Maeyypa0JaeGimaadabaGaeG4mamdaniabggHiLdGccqGHflY1cqWGSbaBdaahaaWcbeqaaiabd6gaUbaakiabcYcaSaaa@429E@

where *l *and *a*_*n *_are the physical distance and the regression coefficients (we used only SNP pairs with distances of 10 k to 100 k bps). We used Akaike's Information Criteria (AIC) to determine that this model was the best fit among several simple (linear, exponential, logarithmic, power, quadratic, or cubic) models.

To adjust the effect of physical distance on LD, we calculated the residual (*r*^2^_*res*_) by subtracting the expected *r*^2 ^value from the observed *r*^2 ^value,

*r*^2^_*res *_= *r*^2 ^- *E*(*r*^2^),

which we regressed with the observed feature proportion,

*r*^2^_*res *_= *cp *+ *d*,

where *p*, *c*, and *d *are the observed proportion, the regression coefficient, and the intercept, respectively. This coefficient was used to compare the influence of each feature on LD (see Additional file 2). We applied this simple regression to each sequence feature instead of multivariate regression, since simple regression is widely considered more effective for interpreting the regression coefficient. In the simple regression, we obtained a further residual for a permutation test. In this test, we randomly shuffled the residuals of conserved and non-conserved categories and obtained a difference between the means of the residuals over the two artificial categories 10,000 times to calculate a *p*-value.

### Partial correlation analysis

We performed a partial correlation analysis to simultaneously evaluate the effects of multiple sequence features on LD, which cannot be attained simply by plotting LD. We used two partial correlation coefficients (*R*_1 _and *R*_2_) between *r*^2 ^and the base-pair proportion (*p*_*cns*_) of conserved regions within SNP pairs, given only physical distance (*l*); and given both physical distance and the proportion (*p*_*feature*_) of each of the sequence features, such as GC, gene, or repeat:

*R*_1_(*r*^2^, *p*_*cns *_| *l*),

*R*_2_(*r*^2^, *p*_*cns *_| *l*, *p*_*feature*_).

If the value of *R*_1 _differed from that of *R*_2_, we attributed the difference to *p*_*feature*_.

### Recombination rates

We downloaded the datasets of fine-scale recombination rates from the HapMap Project [4] website. We calculated the average of recombination rates across specified regions: (Σ*ρ*_*i*_)/*l*, where *l *is the total length of the regions and *ρ*_*i *_is a recombination rate at a base position *i *within the regions.

## Authors' contributions

MK and TaT planned the research. MK performed the analyses and wrote the manuscript. TAJ, ToT, AS, YO, YN, and TaT reviewed the manuscript. All authors read and approved the final manuscript.

## Supplementary Material

Additional File 1 A figure showing plots of the moving average of the fraction of complete or nearly complete LD (*r*^2 ^> 0.8) versus distance between SNPs for all four populations. Click here for file

Additional File 2 A table showing the regression coefficients, ratios of base pairs in conserved regions to non-conserved regions, and base-pair fractions in the genome for sequence features. Click here for file

Additional File 3 A table showing the results of partial correlation analysis to detect sequence features that involve weaker LD in conserved regions. Click here for file

## Figures and Tables

**Table 1:** Recombination rates for highly and less highly conserved regions

	Recombination rate (cM/Mb)
Highly conserved regions	2.05
Less highly conserved regions	2.24
Highly conserved non-coding regions	2.54
Less highly conserved non-coding regions	2.98
Highly conserved coding rich-regions	0.62
Less highly conserved coding rich-regions	1.46
Small genes	1.78

These regions correspond to the regions in Figure 1D, in which we used highly and less highly conserved regions, highly and less highly non-coding conserved regions, and regions enriched (>20% in the bases) with highly and less highly conserved coding regions. As in Figure 1D, we selected only regions where the proportion of repeats was <20%, and since after this adjustment we found outliers of LD related to extreme GC-content, we further selected regions where the GC-content was 45–65%. For reference, we list the recombination rate in small genes with sizes up to 1000 bps and with the same conditions as to repeat proportion and GC-content. The average recombination rate in the genome was 1.33 cM/Mb.
