# Supplementary material for: Linkage disequilibrium of evolutionarily conserved regions in the human genome
Source: BMC Genomics. 2006 Dec 28;7:326. doi: 10.1186/1471-2164-7-326 (PMC1769491; doi:10.1186/1471-2164-7-326)

Additional file 1 - A moving average of the fraction of complete or nearly complete LD ($r^2$>0.8) versus distance between SNPs. (A) Plots of LD within DNA sequences conserved between the human and mouse genomes (in red with Xs), non-conserved regions (regions other than conserved ones; shown in red with circles), genic regions (in blue with Xs), and non-genic regions (in blue with circles).

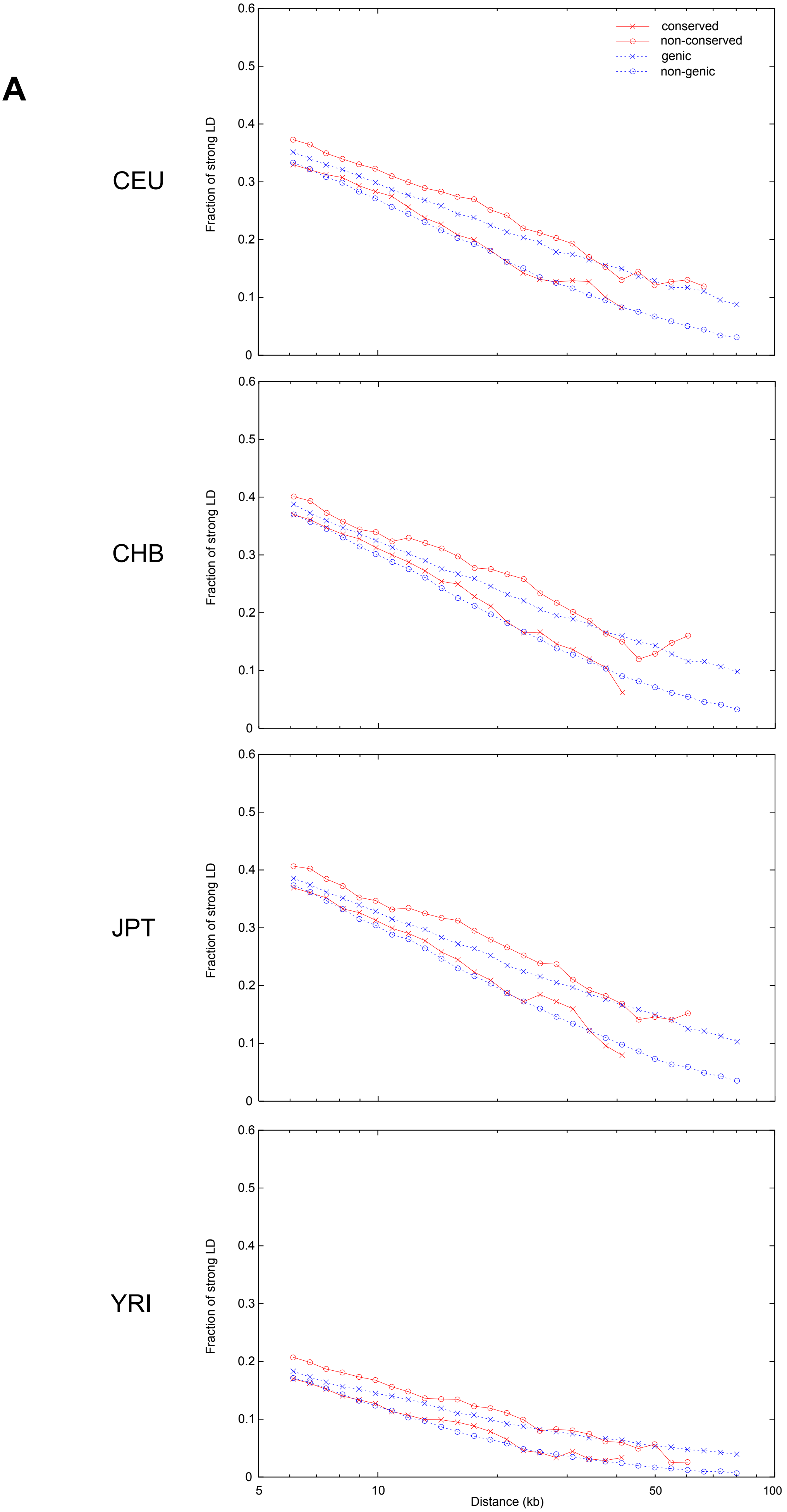

(B) Plots of LD within intersections of non-genic regions with conserved (in red with Xs) and non-conserved (in red with circles) regions, and of genic regions with conserved (in blue with Xs) and non-conserved (in blue with circles) regions.

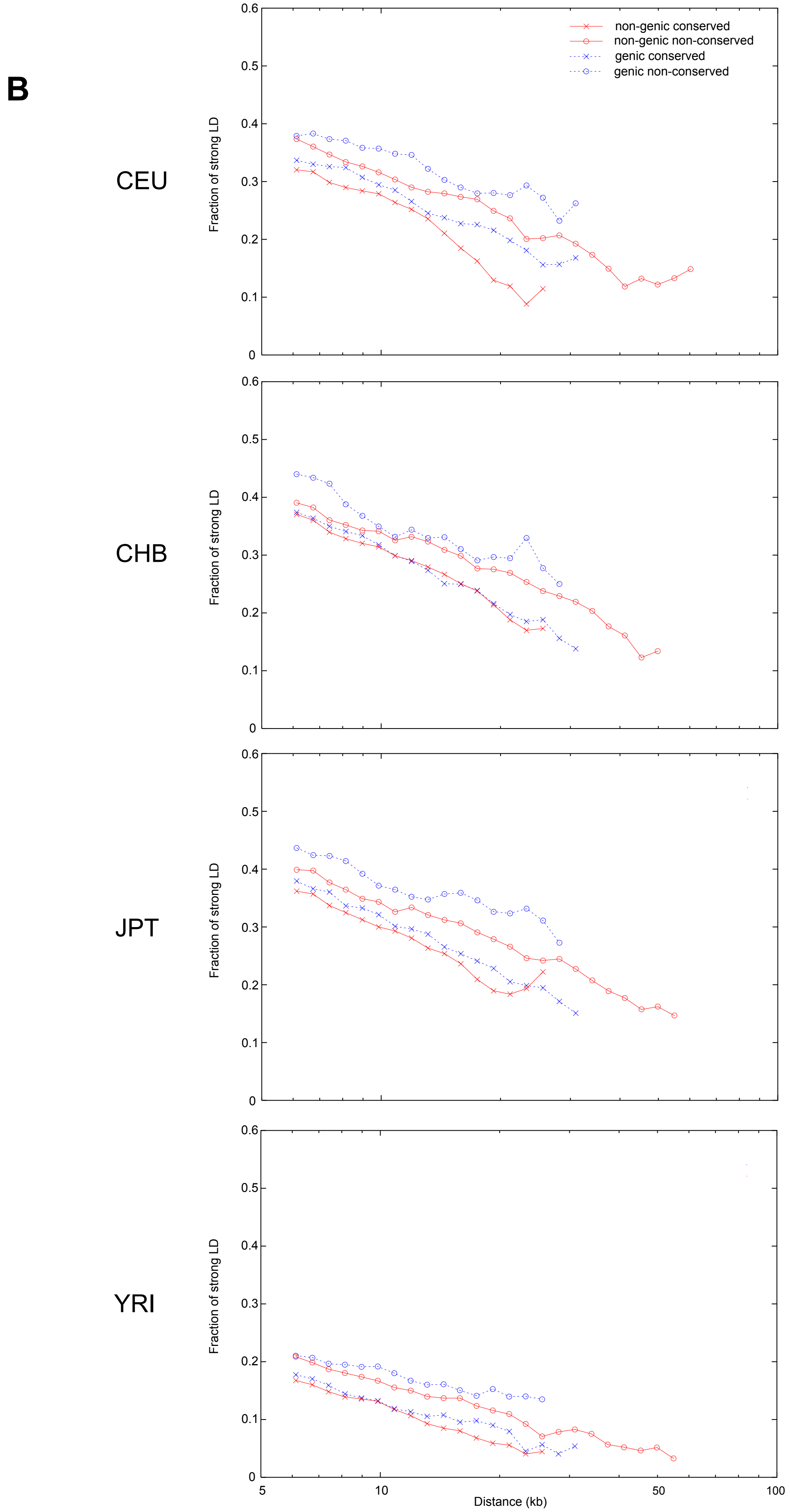

(C) Plots of LD within intersected regions of centromeric regions (the 10% definition, we only show plots in the 10% definition because of the same tendency in the 5% definition) with conserved (in red with Xs) and non-conserved (in red with circles) regions, of telomeric regions with conserved (in blue with Xs) and non-conserved (in blue with circles) regions, and of the residual regions (neither centromeric nor telomeric) with conserved (in green with Xs) and non-conserved (in green with circles) regions.

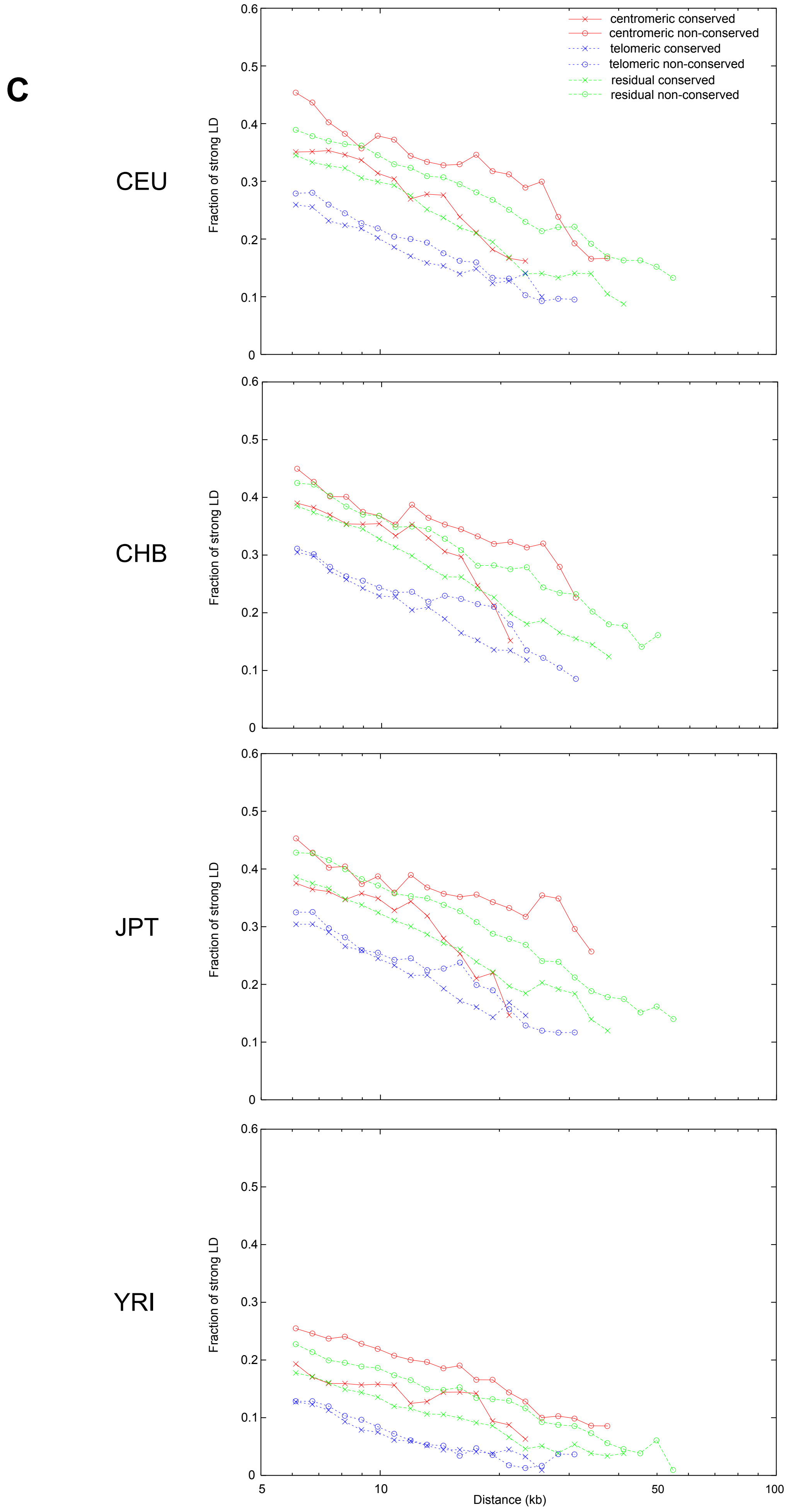

(D) LD fractions for SNP pairs within highly conserved and less highly conserved regions (black and green), highly and less highly conserved non-coding regions (blue and light blue), and regions enriched (>20% in the bases) with highly and less highly conserved coding regions (red and pink). We selected only regions where the GC-content was 45-65% and the proportion of repeats was <20%.

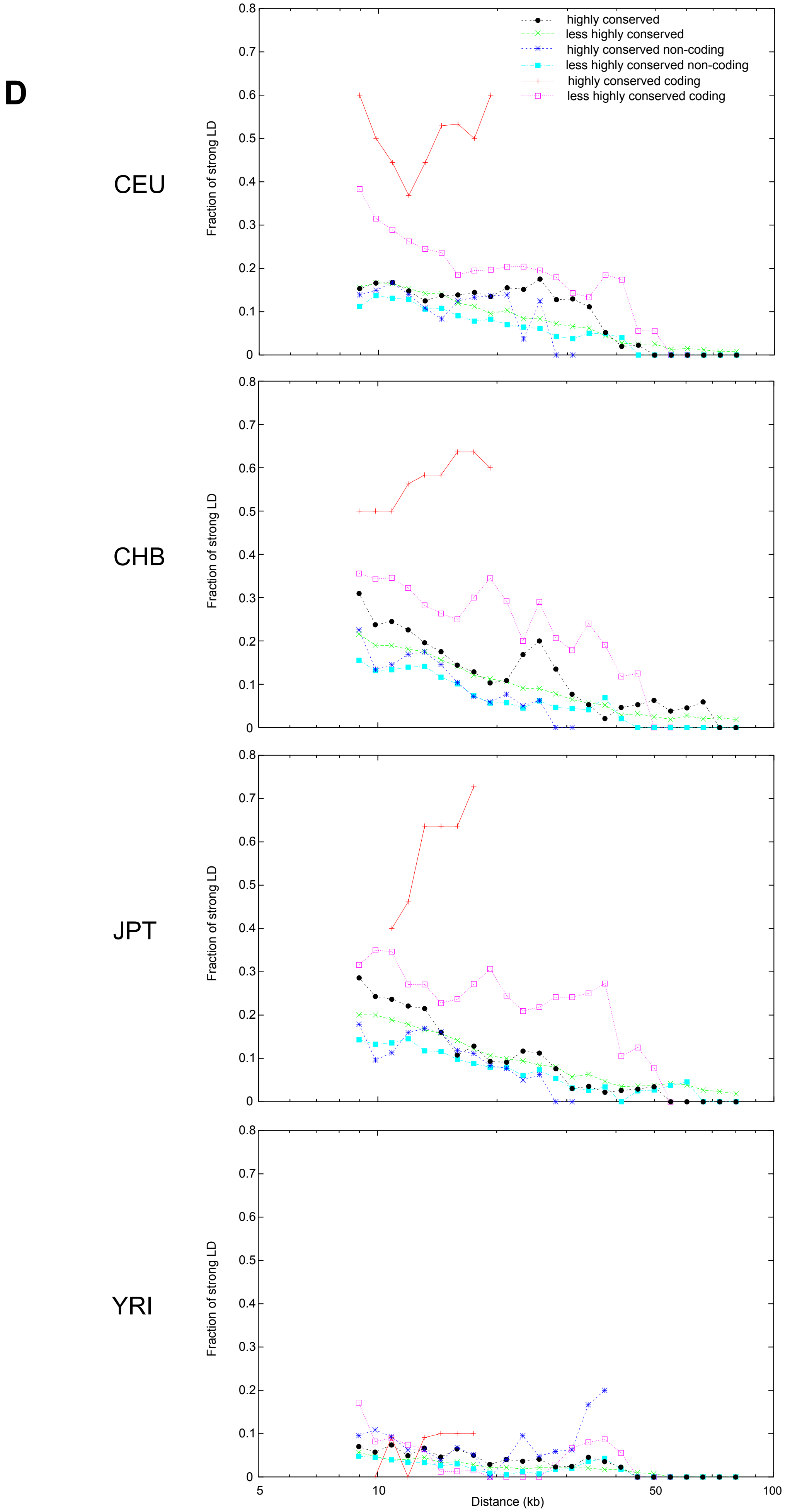

Supplement: Additional File 1 — A figure showing plots of the moving average of the fraction of complete or nearly complete LD (r2 > 0.8) versus distance between SNPs for all four populations. [file 1471-2164-7-326-S1.pdf]
