# Supplementary material for: Linkage disequilibrium of evolutionarily conserved regions in the human genome
Source: BMC Genomics. 2006 Dec 28;7:326. doi: 10.1186/1471-2164-7-326 (PMC1769491; doi:10.1186/1471-2164-7-326)
Supplement: Additional File 2 — A table showing the regression coefficients, ratios of base pairs in conserved regions to non-conserved regions, and base-pair fractions in the genome for sequence features. [file 1471-2164-7-326-S2.doc]

Additional file 2

Regression coefficients, ratios of base pairs in conserved regions to non-conserved regions, and base-pair fractions in the genome for sequence features

|  | Regression coefficient | | | | Ratio in conserved  to non-conserved regions | Base fraction in  genome (%) |
| --- | --- | --- | --- | --- | --- | --- |
|  | CEU | CHB | JPT | YRI |  |  |
| GC | -1.14 | -1.17 | -1.16 | -0.72 | 1.02 | 41.0 |
| CpG | -3.31 | -3.37 | -3.32 | -2.03 | 1.08 | 5.3 |
| All repeat | 0.25 | 0.26 | 0.26 | 0.18 | 0.48 | 44.6 |
| SINE/Alu | 0.19 | 0.22 | 0.22 | 0.23 | 0.66 | 10.7 |
| SINE/MIR | -1.98 | -2.01 | -2.06 | -1.34 | 1.14 | 2.9 |
| LINE/L1 | 0.51 | 0.52 | 0.53 | 0.36 | 0.32 | 17.4 |
| LINE/L2 | -0.36 | -0.39 | -0.41 | -0.31 | 0.49 | 3.3 |
| LTR/ERV1 | 0.10 | 0.08 | 0.11 | 0.02 | 0.38 | 2.9 |
| LTR/MaLR | -0.38 | -0.47 | -0.47 | -0.34 | 0.47 | 3.8 |
| Satellite | 0.34 | 0.22 | 0.32 | 0.28 | 0.05 | 0.3 |

Regression coefficients were calculated on the model of *r^2^* adjusted by the physical distance and the proportion of bases contained in each sequence feature within a SNP pair. All the coefficients were significant (*p*<10^-15^) by the *t*-test. “Ratio” means a ratio of the proportion of total bases in each sequence feature within conserved regions to that within non-conserved ones.
