# Supplementary material for: Linkage disequilibrium of evolutionarily conserved regions in the human genome
Source: BMC Genomics. 2006 Dec 28;7:326. doi: 10.1186/1471-2164-7-326 (PMC1769491; doi:10.1186/1471-2164-7-326)
Supplement: Additional File 3 — A table showing the results of partial correlation analysis to detect sequence features that involve weaker LD in conserved regions. [file 1471-2164-7-326-S3.doc]

Additional file 3

Partial correlation analysis to detect sequence features that involve weaker LD in conserved regions

CEU

|  | *R_1_* | *R_2_* | | | | |
| --- | --- | --- | --- | --- | --- | --- |
|  | - | CpG | GC | Exon | Gene | Repeat |
| Conserved region | -0.053 | -0.042 | -0.035 | -0.061 | -0.072 | 0.008 |
| Exon | 0.036 | - | - | - | - | - |

CHB

|  | *R_1_* | *R_2_* | | | | |
| --- | --- | --- | --- | --- | --- | --- |
|  | - | CpG | GC | Exon | Gene | Repeat |
| Conserved region | -0.049 | -0.039 | -0.032 | -0.059 | -0.069 | 0.011 |
| Exon | 0.045 | - | - | - | - | - |

JPT

|  | *R_1_* | *R_2_* | | | | |
| --- | --- | --- | --- | --- | --- | --- |
|  | - | CpG | GC | Exon | Gene | Repeat |
| Conserved region | -0.054 | -0.044 | -0.038 | -0.064 | -0.074 | 0.006 |
| Exon | 0.047 | - | - | - | - | - |

YRI

|  | *R_1_* | *R_2_* | | | | |
| --- | --- | --- | --- | --- | --- | --- |
|  | - | CpG | GC | Exon | Gene | Repeat |
| Conserved region | -0.046 | -0.037 | -0.032 | -0.057 | -0.065 | 0.013 |
| Exon | 0.051 | - | - | - | - | - |

The second column (*R_1_*) indicates a partial correlation coefficient between *r^2^* and the proportion of bases contained in conserved regions or exons (for reference) within SNP pairs, given physical distance within the pairs. The absolute value of the coefficient for conserved regions appears somewhat larger than that for exons. The third or later column (*R_2_*) indicates a partial correlation coefficient between *r^2^* and the proportion of bases contained in conserved regions within SNP pairs, given both physical distance within the pairs and the proportion of bases contained in each sequence feature, such as CpG, GC, exon, gene, and repeat. In only the case of repeat, the negative correlation between *r^2^* and the proportion of conserved regions vanishes. All correlations are significant (*p*<10^-16^) by the *t*-test.
